# A Brain-Inspired Adaptive Space Representation Model Based on Grid Cells and Place Cells

**DOI:** 10.1155/2020/1492429

**Published:** 2020-08-11

**Authors:** Kun Han, Dewei Wu, Lei Lai

**Affiliations:** Information and Navigation College, Air Force Engineering University, Xi'an, Shaanxi 710077, China

## Abstract

Grid cells and place cells are important neurons in the animal brain. The information transmission between them provides the basis for the spatial representation and navigation of animals and also provides reference for the research on the autonomous navigation mechanism of intelligent agents. Grid cells are important information source of place cells. The supervised learning and unsupervised learning models can be used to simulate the generation of place cells from grid cell inputs. However, the existing models preset the firing characteristics of grid cell. In this paper, we propose a united generation model of grid cells and place cells. First, the visual place cells with nonuniform distribution generate the visual grid cells with regional firing field through feedforward network. Second, the visual grid cells and the self-motion information generate the united grid cells whose firing fields extend to the whole space through genetic algorithm. Finally, the visual place cells and the united grid cells generate the united place cells with uniform distribution through supervised fuzzy adaptive resonance theory (ART) network. Simulation results show that this model has stronger environmental adaptability and can provide reference for the research on spatial representation model and brain-inspired navigation mechanism of intelligent agents under the condition of nonuniform environmental information.

## 1. Introduction

Environmental cognitive ability is the basis of free movement of animals and intelligent agents. Learning from nature and brain is an important method to study the autonomous navigation mechanism of intelligent agents [1]. The hippocampal structure in the brain is an important organization related to episodic memory and spatial navigation and is the core area that constitutes the neural circuit of cognitive map. The hippocampal structure contains a variety of cells which are related to spatial representation and located in different regions, such as place cells [2], grid cells [3], head-direction cells [4], and boundary vector cells [5]. Through information transformations between these cells, spatial representation [6], cognitive map construction [7, 8], goal navigation [9, 10], episodic memory [11],and other functions can be realized.

Place cells and grid cells represent space in different ways. Place cells are mainly located in the hippocampus CA1, CA3, and dentate gyrus. In familiar environment, place cell has a single or limited number of firing fields. When an animal conducts spatial exploration, a certain number of place cells randomly constitute cell population to realize space representation [12]. The changes of the environment may cause global remapping [13, 14], partial remapping [15], or firing rate remapping [16, 17] of the place cell population. Grid cells are mainly located in the entorhinal cortex, which includes the middle entorhinal cortex and the lateral entorhinal cortex and is an important information source of hippocampus. Grid cell has regular hexagonal firing field extending to the whole space, which is characterized by size, spacing, phase, and direction. The grid cells with similar firing field spacing and direction are clustered into cell module. The ratios of firing field spacing between any adjacent modules are similar [18–21]. Self-motion information is an important information source of grid cells to maintain the firing field stability [22–24]. However, the firing field phase and direction may be varied with the change of environment [25–27].

Grid cells are important information source of place cells [28–31]. Since grid cells were discovered, researchers have proposed a variety of generation models of place cells from grid cell inputs. In the unsupervised models, the place cells are generated through the weighted summation of the grid cell inputs and the weights from grid cells to place cells are trained through competition mechanism [32–34]. In the supervised models, the visual place cells are generated from environment information and are used as supervision to update the weights from grid cells to place cells [35–37]. Although the existing models have simulated the generation of place cells from grid cell inputs, there still exists shortcoming. In these models, the grid cells are generated from self-motion information. The firing models of grid cell driven directly by the self-motion information can be divided into the continuous attractor network model [38] and the oscillatory interference model [39]. The continuous attractor network model is based on the preset activation-inhibition connections between grid cells, namely, local activation and long-range inhibition. The parameters in oscillatory interference model include maximum firing rate, firing field spacing, firing field direction, and firing field phase and are also preset [32–34]. Therefore, the firing characteristics of grid cell and place cell cannot adapt to the environment.

When the first outbound exploration of the rat pups, place cells and grid cells develop simultaneously [7, 30, 40]. It is suggested that there may exist information transformations between place cells and grid cells. In this paper, we propose a united generation model of grid cells and place cells which has the ability to adapt to the environment. In order to distinguish all kinds of grid cells and place cells, the place cells generated from environment information are called visual place cells, the grid cells generated from visual place cell inputs are called visual grid cells, the grid cells generated from self-motion information are called self-motion grid cells, the grid cells generated from two information sources are called united grid cells, and the place cells generated through supervised learning are called united place cells. In this model, the generation process of united grid cells and united place cells is mainly divided into three steps. First, visual place cells generate visual grid cells along the boundary through feedforward network. Second, visual grid cells and self-motion information generate united grid cells extending to the whole space through genetic algorithm. Third, united grid cells generate more compact united place cells in the sparse area of visual place cells through supervised fuzzy ART network. The model can be used for the spatial representation of intelligent agents.

## 2. Models

**Figure 1:**
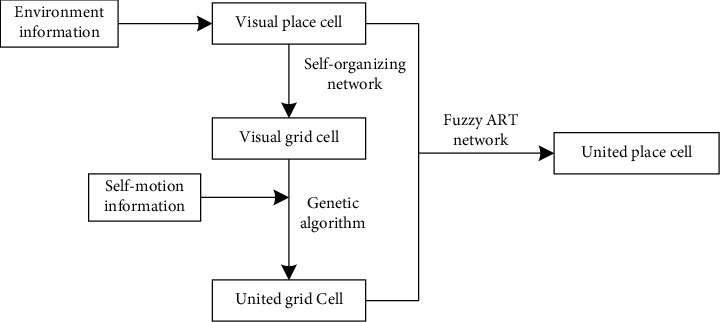
The generation process of united grid cells and united place cells.

Visual place cells are driven by the external environment and own high stability, absolute location information, and the earlier generation time. Therefore, they are used as the supervisions for the generation of united grid cells and united place cells. The generation process of united grid cells and united place cells is shown in Figure 1.

### 2.1. Visual Place Cells Generate Visual Grid Cells

It is assumed that the agent explores a rectangular space at the speed *v* and reaches any location with the same probability. The spatial boundary information drives the generation of visual place cells which have a tighter distribution near the boundary. Gaussian function is used to represent the firing field of visual place cell:

(1) $$\begin{array}{c} R_{i}\left(x,y\right)=A\;\exp\left\{-\frac{\left[{\left(x-x_{i}\right)}^{2}+{\left(y-y_{i}\right)}^{2}\right]}{2\sigma_{i}^{2}}\right\}, \end{array}$$

where *R*_*i*_(*x*, *y*) is the firing rate of the *i*‐th visual place cell at the location (*x*, *y*); *A* is the maximum firing rate of visual place cells; (*x*_*i*_, *y*_*i*_) is the place where the *i*‐th visual place cell is generated.

When the agent explores freely, if the firing rates of all visual place cells were less than $A/\sqrt{2}$, new visual place cell will be generated. *σ*_*i*_ is the standard deviation of firing field size of the *i*‐th visual place cell, which increases as *d* goes up:

(2) $$\begin{array}{c} \sigma \left(d\right)=l_{\min}^{\left(L-d\right)/\left(L-1\right)}l_{\max}^{\left(d-1\right)/\left(L-1\right)},\;d\leq D, \end{array}$$

where *d* is the minimum distance from the exploring location to the boundary; *l*_min_ is the minimum standard deviation of the firing field size; *l*_max_ is the maximum standard deviation of the firing field size; *L* is the firing field distribution constant; *D* is the maximum distance from which visual place cells can be generated.

The feedforward network based on place cell inputs and Hebbian learning to weights can be used to generate grid cell with hexagonal firing field [41–46]. The periodic grid cell firing field is derived from the periodic weight distribution from place cells to single grid cell, and the input correlation driving the development of periodic weight distribution is usually presented as the Mexican hat model. The input correlation with Mexican hat model may be derived from the temporal correlation [42–44] or the spatial correlation [45, 46] of the place cell firing rates. However, the existing temporal correlation models assume the Hebbian learning as nonlinear correlation plasticity [42], the spiking rate adaptive function as Mexican hat model [43], or the weight window function as Mexican hat model [44]. We found that, without any presupposition, the input correlation with Mexican hat model can be derived only through the linear temporal correlation of the place cell firing rates. The firing field spacing of grid cell generated by this model is proportional to the exploring speed of the intelligent agent. It is assumed that the weight update from the place cell population to a grid cell has a certain time interval. The Hebbian learning is implemented based on the change of place cell firing rates before and after the time interval and the real-time grid cell firing rate. The weight update can be expressed as follows:

(3) $$\begin{array}{c} \eta^{-1}\frac{\text{d}E_{n}}{\text{d}t}=-E_{n}+\left[R_{n}^{\text{visual}}\left(t\right)-hR_{n}^{\text{visual}}\left(t-\tau \right)\right]G^{\text{visual}}\left(t\right)+S\\ =-E_{n}+\sum_{i}\left[R_{n}^{\text{visual}}\left(t\right)-hR_{n}^{\text{visual}}\left(t-\tau \right)\right]R_{i}^{\text{visual}}\left(t\right)E_{i}+S, \end{array}$$

where *E*_*n*_ is the weight from the *n*‐th visual place cell to the generated visual grid cell; *η* is weight update rate; *τ* is the weight update time interval; *h* is the reduction coefficient of place cell firing rate; *G*^visual^(*t*)=∑_*i*_*E*_*i*_*R*_*i*_^visual^(*t*) is the firing rate of visual grid cell generated from visual place cell inputs at any moment *t*; *S* is the weight update constant.

In order to develop weights with periodic spatial distribution, competitive nonlinear restriction is applied. The upper boundary *Th*_up_ and the lower boundary *Th*_down_ of the weights are set, respectively. When a weight is less than the lower boundary, the weight is set to the lower boundary. When any weight is larger than the upper boundary, all weights are equally scaled down through competition, so that the maximum weight is equal to the upper boundary.

### 2.2. Visual Grid Cells and Self-Motion Information Generate United Grid Cells

In the existing models, either place cell inputs or self-motion information can generate grid cells independently. However, in this paper, on the one hand, since the firing field distribution of visual place cells varies with the change of the distance from the exploring location to the boundary, the firing field of visual grid cell generated from the visual place cell inputs through the feedforward network cannot expand to the whole space. On the other hand, the firing field parameters of self-motion grid cells generated from self-motion information need to be preset and cannot be adaptive to the environment. In view of the above shortcomings, we combine the visual grid cell and the self-motion information through genetic algorithm to generate the united grid cell with firing field adaptive to environment and extending to the whole space.

The grid cell models driven directly by self-motion information mainly include continuous attractor network model [38] and oscillatory interference model [39]. The continuous attractor network model represents the firing pattern of the grid cell population. The asymmetrical intercellular connections and self-motion information make the firing pattern move as a whole. The oscillatory interference model represents the firing rate of a single grid cell. The self-motion information causes the phase shift of each oscillator, so as to change the firing rate. In this paper, the united grid cells are independent of each other and there is no interconnection. Therefore, the united grid cell is represented by the oscillatory interference model referring to [34]. The firing rate of united grid cell at location **r**=(*x*, *y*) can be expressed as

(4) $$\begin{array}{c} \left\{\begin{array}{c} G^{\text{self}}\left(r\right)=C*\frac{2}{3}\left\{\frac{1}{3}\sum_{d=1}^{3}\cos\left[\frac{4\pi }{\sqrt{3}B}k_{d}\left(r-\varphi \right)\right]+\frac{1}{2}\right\},\\ k_{d}={\left[\cos\left(\omega +\frac{2\left(d-1\right)}{3}\pi \right),\;\sin\left(\omega +\frac{2\left(d-1\right)}{3}\pi \right)\right]}^{T}, \end{array}\right. \end{array}$$

where *C* is the maximum firing rate; *B* is the firing field spacing; *φ*=[*x*_0_, *y*_0_] is the firing field phase; *ω* is the firing field direction.

The visual grid cell whose firing characteristics are adaptive to environment is generated from visual place cell inputs. We regard the firing field of visual grid cell as the sample of the firing field of united grid cell along one certain environmental boundary. The genetic algorithm is used to optimize the parameters in (4) to maximize the similarity between the firing characteristics of visual grid cell and the firing characteristics of united grid cell. The parameters optimization can be seen as where the grid pattern which is adaptive to environment comes.

**Figure 2:**
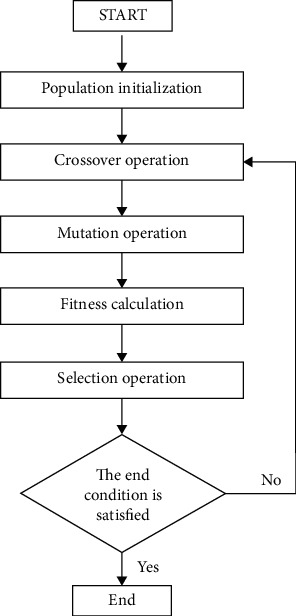
The genetic algorithm.

Genetic algorithm is a model to search for the optimal solution by simulating the biological evolution process. It begins with populations that represent the potential set of solutions to a problem. After the initial populations, according to the principle of survival of the fittest, generation evolution produces better approximate solutions. In each generation, crossover and mutation are performed with the help of genetic operators to generate new populations representing a new solution set, and then populations are selected according to the fitness. This process will result in having selected populations more adaptive to the environment than the populations in previous generation. The optimal population in the last generation is regarded as the approximate optimal solution. The genetic algorithm can be shown in Figure 2.

In this paper, set the update range of the parameters as *C* ∈ [0,1], *B* ∈ [0, min(*L*1, *L*2)/2], *x*_0_ ∈ [0, *L*1], *y*_0_ ∈ [0, *L*2], and *ω* ∈ [0,2*π*]. The evolution process of genetic algorithm is as follows.

① Initialize the population randomly. The population size is *N*; each population contains the above five parameters; each parameter is represented by *M* bits binary.
② Set crossover probability *pc* and mutation probability *pm*. *N* offsprings are generated through crossover operator and mutation operator.
③ Record the firing rate *G*^visual^(*k*) of visual grid cell and calculate the firing rates *G*^united^(*k*, 1 ~ 2*N*) of united grid cell in the sampling region. When the record number *k* reaches *K*, the fitness of each population is calculated. The fitness is defined as the quadratic sum of the firing rate differences at each record moment; namely, FitValue(1 ~ 2*N*)=∑_*k*=1_^*K*^[*G*^visual^(*k*, 1 ~ 2*N*) − *G*^united^(*k*)]^2^.
④ Select *N* populations with low fitness from the parent and the offspring as the next generation.
⑤ Record the optimal solution and reset the record number *k* to zero.
⑥ Determine whether the end condition is satisfied. If so, output the optimal solution; if not, return to step ②.

### 2.3. Visual Place Cells and United Grid Cells Generate United Place Cells

Influenced by the boundaries, the visual place cells have nonuniform distribution. However, the grid cells can generate place cells by competitive neural network whose parameters can influence the firing field characteristics of the generated place cells. Therefore, the combination of visual place cells and united grid cells can improve the distribution density and positioning accuracy of the place cells far away from the boundaries. In this paper, supervised fuzzy ART network is used to realize the information transmission from the visual place cells and united grid cells to the united place cells.

**Figure 3:**
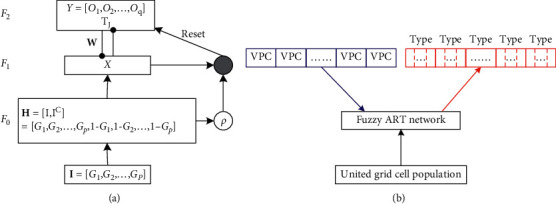
(a) The fuzzy ART network structure. (b) The supervised fuzzy ART network structure. VPC represents the visual place cell.

ART network is a competitive classifying and clustering network with both plasticity and incremental learning. It has the ability of learning new knowledge and meanwhile maintaining the memory of old knowledge. Therefore, the learning process is robust to the input order of the samples. ART network mainly includes ART1 network for binary input processing, ART2 network for real input processing [47], ART3 network for multilayer network [48], fuzzy ART network for fuzzy processing [49], and ARTMAP network for supervised learning [50, 51]. The fuzzy ART network structure is shown in Figure 3(a).

The competitive learning of fuzzy ART network includes the following steps.

① Input preprocessing: **I**=[*G*_1_, *G*_2_,…, *G*_*p*_] is the normalized input vector, and the range of each element *G*_*i*_ is [0,1]; the parameter *p* represents the number of input elements; **H**=[**I**, **I**^*C*^] is the complement representation of the input vector.
② Category selecting: for the input vector **H** and the node *O*_*j*_ in field *F*_2_, the selection function *T*_*j*_ is defined as
  (5) $$\begin{array}{c} T_{j}\left(H\right)=\frac{\left|H\wedge w_{j}\right|}{\alpha +\left|w_{j}\right|},\;j=1,2,\ldots ,q, \end{array}$$
  where *α* is a small nonnegative real, and the value in this paper is 0.001; *q* is the number of nodes in field *F*_2_; **w**_*j*_ is the adaptive weight vector from input vector **H** to node *O*_*j*_, and the initial value of each weight is 1; ∧ is the fuzzy sum operator defined as (*U*∧*V*)_*i*_ ≡ min(*u*_*i*_, *v*_*i*_); |·| is 1-norm defined as |*U*| ≡ ∑_*i*_|*u*_*i*_|.
The node *O*_*J*_ corresponding to the largest function *T*_*J*_(**H**) in all the selection functions is regarded as the category. If there are multiple maximum selection functions at the same time, the node with the smallest index is selected as the category. After the category selection, the vector **X** in field *F*_1_ is calculated:
  (6) $$\begin{array}{c} X=\left\{\begin{array}{cc} H, & \text{no node is selected,}\\ H\wedge w_{J}, & \text{node }O_{J}\text{ is selected.} \end{array}\right. \end{array}$$
③ Category matching: to match **X** and **H**, if |**X**| ≥ *ρ*|**H**|, the match succeeds; otherwise, the match fails. *ρ* ∈ [0,1] is the match parameter. If the match fails, the selection function *T*_*J*_(**H**) will be set to zero, and the learning will return to step ② to select category and match category again. The match process will end until the match succeeds or all *q* nodes in field F_2_ have been tried.
④ Weight updating: if the input vector **H** matches the node *O*_*J*_ successfully, the weight vector **w**_*J*_ will be updated. If the input vector **H** does not match any node in field F_2_, a new node will be added as the match node, and the weight vector from the input vector to the added node is initialized. The update of weight vector is expressed as
  (7) $$\begin{array}{c} w_{J}^{\text{new}}=\beta \left(H\wedge w_{J}^{\text{old}}\right)+\left(1-\beta \right)w_{J}^{\text{old}}, \end{array}$$
  where *β* ∈ [0,1] is the learning rate. When *β*=1, the process is defined as fast learning. In this paper, we take *β*=1.
In the existing ART network models, the supervised network is ARTMAP network which includes a pair of fuzzy ART networks (i.e., ART_a_ and ART_b_). The ART_b_ network provides learning supervision for the ART_a_ network. The process of generating united place cells is actually to classify firing rates of united grid cell population. In the model of generating united place cells from visual place cells and united grid cells, we simplify the supervised ARTMAP network. According to the firing fields of visual place cells, the whole space is divided into different types, between which there may exist overlap. The firing field of each visual place cell is one type, and the region without visual place cells is one type. The ART_b_ network is replaced by the visual place cell types as the supervision of the ART_a_ network, and the input vector of the ART_a_ network is the firing rates of united grid cell population. Each type is divided into a number of categories which are defined as the united place cells. This simplification enables the fuzzy ART network to have the supervised learning ability. The supervised fuzzy ART network structure is shown in Figure 3(b).
In Figure 3(b), the inputs are the firing rates of united grid cell population and the fuzzy ART network is the structure in Figure 2(a). The blue blocks represent the visual place cells which act as supervisors. Their firing fields are small enough that one type contains only one category. They are used to train the parameters in the fuzzy ART network. The red blocks represent the types which are divided into different categories. They include the visual place cells whose firing fields are large enough and the region where there is no visual place cell. The category range, namely, the firing field size of the united place cell, is determined by the trained parameters in the fuzzy ART network.

## 3. Results

### 3.1. The Firing Field of Visual Grid Cell Distributes Periodically along the Boundary

The environment and boundary information drive the generation of visual place cells with different distribution density, and then visual place cells generate locally distributed visual grid cell through feedforward network. Simulation parameters of visual grid cell are shown in Table 1.

**Figure 4:**
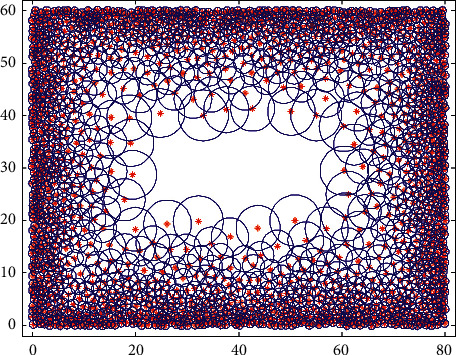
The distribution of visual location cells. After free space exploration, a total of 1487 visual place cells are generated. The red marks indicate the locations where the visual place cells are generated, and the blue circles indicate the region with firing rate $A/\sqrt{2}$.

The agent explores the whole space and reaches any location with the same probability. According to the generation process of visual place cells introduced in Section 2.1, after 1*e*5 s exploration, the distribution of visual place cells is shown in Figure 4.

As can be seen from Figure 4, in the region close to the boundaries, the distribution of visual place cells is closer and the firing field size is smaller, which suggests a more accurate spatial representation. In the region moving away from the boundaries, the firing field spacing and size increase gradually, and the spatial representation becomes fuzzy. The distribution of visual place cells conforms to the distribution characteristics of initial place cells proposed in the preweaning rat experiment [30].

**Figure 5:**
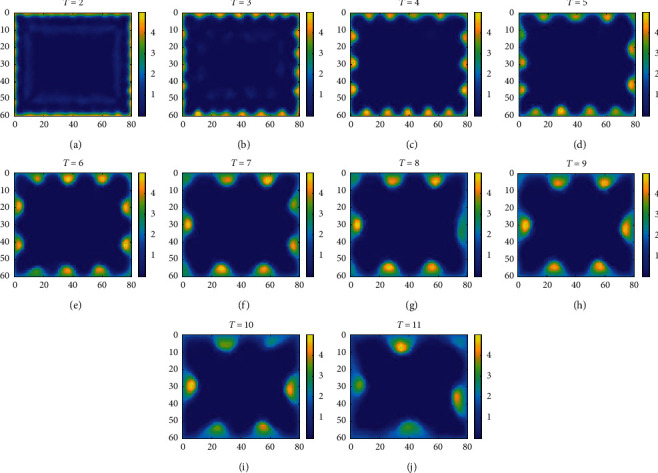
The weights from visual place cell to visual grid cell learned under different weight update time intervals. The parameter on the top of each image is the weight update time interval *τ*. The color bar represents the weight.

In the brain, the appearance of mature grid cell is later than that of mature place cell. Therefore, it is suggested that the place cells can provide input information for the generation of grid cells. Assuming that the weight update time interval *τ* in this paper is a positive integer, the change of *τ* will have an influence on the weight distribution under the same exploring speed. Taking the visual place cells shown in Figure 4 as the information source of the visual grid cell and according to the weight update model introduced in Section 2.1 and the parameters in Table 1, the weights from visual place cell to visual grid cell learned under different weight update time intervals are shown in Figure 5.

The weights and the firing field of visual grid cell have the same distribution. Therefore, it can be seen from Figure 5 that the firing field of visual grid cell is influenced by the weight update time interval and boundary. When the time interval is small (e.g., *τ*=2), the visual grid cell with periodic firing field cannot be generated in the rectangular space. In fact, this is because the small time interval does not make the weight update process ((3)) meet the reaction-diffusion mechanism [52]. With the increase of time interval, the visual grid cell with periodic firing field is generated along the boundary and the firing field spacing increases monotonically. Under the same time interval, the boundary influences the firing field distribution of visual grid cell, and the firing field along each boundary can correspond to an independent visual grid cell (e.g., *τ*=5). As the time interval increases continuously, the firing field of generated visual grid cell will gradually lose the periodicity and meanwhile lose the ability of serving as the sample of the united grid cell.

### 3.2. The Firing Field of United Grid Cell Can Extend to the Whole Exploring Space

**Figure 6:**
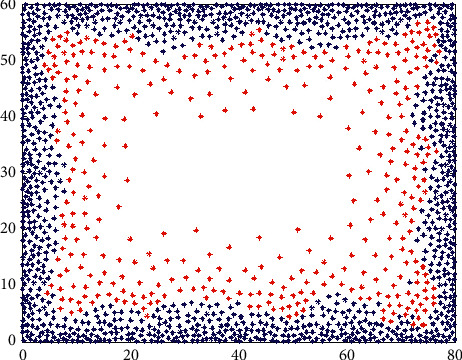
The sampling region of the genetic algorithm. A total of 1088 visual place cells marked in blue are in the sampling region of the genetic algorithm. The other 399 visual place cells marked in red are not in the sampling region of genetic algorithm.

Although the firing field of visual grid cell cannot cover the whole exploring space, it can be used as the sample of united grid cell whose firing field can extend freely. First, the sampling region of the genetic algorithm is determined. If there is activated visual place cell with weight to any visual grid cell greater than threshold Ψ at a certain exploring location, the firing rates *G*^visual^(*k*) and *G*^united^(*k*, 1 ~ 2*N*) are sampled. The simulation parameters of the genetic algorithm used to generate united grid cells are shown in Table 2, and the sampling region of the genetic algorithm is shown in Figure 6.

**Figure 7:**
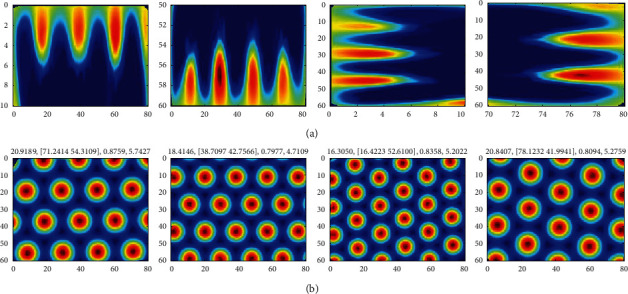
The firing field of generated united grid cell. (a) The firing field of visual grid cell along each boundary when the weight update time interval is *τ*=5. In each image, the maximum firing rate is normalized to 1 Hz, and red represents higher firing rate. (b) The firing field of united grid cell generated based on the corresponding visual grid cell in (a). The parameters on the top of each image are firing field spacing, firing field phase, maximum firing rate, and firing field direction, respectively.

According to the simulation in Section 3.1, the visual grid cells generated when the weight update time interval is *τ*=4 : 7 are selected for the generation of united grid cells through the genetic algorithm. Each visual grid cell independently participates in the generation of a united grid cell, so that four united grid cells could be generated at each time interval. The firing rate of each visual grid cell is normalized so that its maximum firing rate in the sampling region is 1 Hz. The agent explores the region near the boundaries at the speed *v*=2 m/s for 1*e*5 s. According to the evolution process of genetic algorithm introduced in Section 2.2, the firing parameters of the united grid cells are updated. After the exploration, taking the time interval *τ*=5 as an example, the firing fields of the generated four united grid cells are shown in Figure 7.

It can be seen from Figure 7 that the united grid cell generated through genetic algorithm has hexagonal firing field extending to the whole exploring space. And in the sampling region near each boundary the firing field of visual grid cell is almost the same as that of the generated united grid cell. Therefore, the united grid cell generated through genetic algorithm has the characteristics of free expansion and environmental adaptation and is more suitable for spatial representation than the grid cells generated from a single information source.

**Figure 8:**
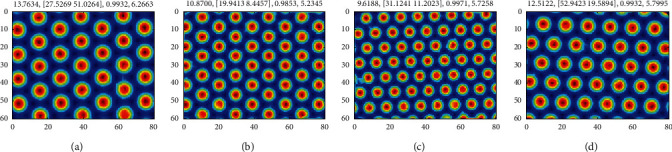
The firing field of generated united grid cell under the preset firing field direction. Each united grid cell corresponds to one visual grid cell in Figure 7(a). The parameters on the top of each image are firing field spacing, firing field phase, maximum firing rate, and firing field direction, respectively.

In Figure 5, because the firing field of each visual grid cell is a one-dimensional distribution along one boundary, the unique united grid cell with hexagon firing field cannot be determined. Further, in view of the above simulation results, the firing field direction is increased *π*/6 as a preset parameter and the other parameters are taken as the learning parameters to conduct space exploring and genetic algorithm learning again. After the exploration, still taking the time interval *τ*=5 as an example, the firing fields of another generated four united grid cells are shown in Figure 8.

As can be seen from Figures 7 and 8, under the same visual grid cell, the firing fields of generated united grid cells with a direction difference of *π*/6 can both match the firing field of visual grid cell precisely. Therefore, they are both used to represent space in this paper. After the learning through the above two genetic algorithms, 32 united grid cells are generated under the condition of 4 different weight update time intervals, and their firing parameters are shown in Table 3.

### 3.3. The Distribution of United Place Cells Is Closer than That of Visual Place Cells

**Figure 9:**
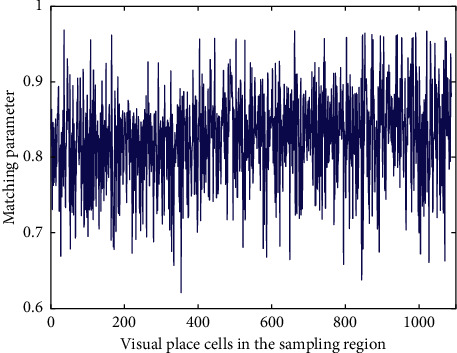
The learning result of matching parameter *ρ*. 1088 matching parameters are learned. The mean value of all matching parameters is 0.8252.

The united grid cells and the visual place cells generate the united place cells through the supervised fuzzy ART network. The united grid cells provide input information, the visual place cells provide supervision information, and the matching parameter *ρ* of the supervised fuzzy ART network determines the distribution density of generated united place cells. In order to make the generated united place cells have uniform distribution density in the whole exploring space similar to that of visual place cells near the boundaries, the matching parameter *ρ* of the supervised fuzzy ART network is learned. The agent explores the space at 0.5 m interval. For the visual place cells satisfying the sampling region of genetic algorithm in Figure 6, the fuzzy ART network is used to implement category learning and real-time adjustment of matching parameter *ρ*, so that there is only one category of united place cell in each type of visual place cell. The learning result of matching parameter is shown in Figure 9.

**Figure 10:**
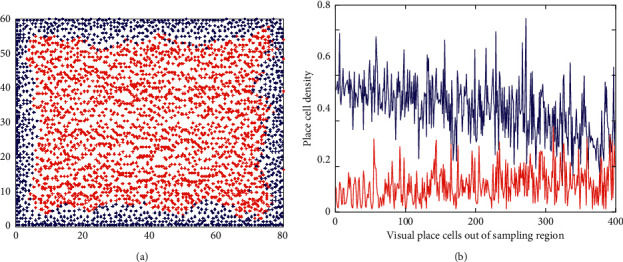
The distribution of generated united place cells. (a) The distribution of united place cells in the whole exploring space. A total of 3,862 united place cells are generated. The blue marks are the united place cells generated in the sampling region of genetic algorithm; the red marks are the united place cells generated in the space that does not belong to the sampling region of genetic algorithm. (b) The place cell density in the firing field of each visual place cell which is not in the sampling region of genetic algorithm. There are a total of 399 visual place cells. The blue solid line indicates the density after category learning; the red solid line indicates the density before category learning.

In Figure 9, each matching parameter ensures that the corresponding visual place cell contains only one category. Different matching parameters are obtained since these visual place cells have different distances to boundary and different firing field sizes. Therefore, the matching parameters are fluctuant. The mean value of all 1088 matching parameters is calculated as the matching parameter of the types that are not in the sampling region of genetic algorithm. The space that does not belong to the sampling region of genetic algorithm is explored successively at 0.5 m interval, and the category learning is implemented for each type based on the supervised fuzzy ART network. The distribution of generated united place cell is shown in Figure 10.

As can be seen from Figure 10, the united place cells generated through supervised fuzzy ART network can not only retain the distribution density of visual place cells near the boundary, but also extend the distribution density to the whole exploring space. Compared with the visual place cells shown in Figure 4, the united place cells are more closely distributed in the region far from the boundary, so the spatial representation accuracy of united place cells is higher.

## 4. Conclusion

Neurons in the hippocampal structure, such as grid cells and place cells, are the basis of environmental cognition and free movement. The research on their firing mechanism can not only deeply understand the working principle of the brain, but also provide reference for the construction of the brain-inspired navigation mechanism of intelligent agents. In this paper, we propose a united generation model of grid cells and place cells, which successively generates visual place cells, visual grid cells, united grid cells, and united place cells. The model can realize the spatial representation and provide a foundation for the construction of navigation cognitive map.

In the generation process of grid cells and place cells, we only presuppose the firing field distribution of visual place cells, and the other three cell types are all the results of environmental adaptation. The visual place cells generate the visual grid cell through feedforward network, whose firing field spacing varies with the change of the weight update time interval. The visual grid cell and self-motion information generate the united grid cell through genetic algorithm, whose firing field extends to the whole exploring space. The visual place cells and the united grid cells generate the united place cells through supervised fuzzy ART network, which are evenly distributed in the whole exploring space. Therefore, compared with the existing models, the model in this paper has stronger environmental adaptability and can adaptively represent the space under the condition of uneven distribution of environment information.

Based on the reaction-diffusion mechanism and weights' Hebbian learning, grid cell can be generated from the place cell inputs. In the existing models, the network parameters are preset, so the firing field of generated grid cell cannot adapt to the environment. In this paper, the input correlation with Mexican hat model is spontaneously generated by the place cell inputs. This method is discussed in a separate paper which has been accepted.

The visual grid cell and self-motion information are combined to generate the united grid cell through genetic algorithm. The firing field of visual grid cell which is regarded as the sample determines the firing parameters of generated united grid cell. In the brain, grid cells exist in the form of module, and the ratio of firing field spacing between any adjacent modules is almost constant. In this paper, the firing fields of generated united grid cells do not show such characteristics, which indicates that the generation of grid cells requires other information sources in addition to the place cell inputs. To generate grid cells based on multiple information sources will be one of our next research contents.

The ARTMAP network is a supervised ART network. It assigns each input to a unique category by gradually increasing the matching parameters of ART_a_ network. In this paper, we simplify the ARTMAP network so as to make fuzzy ART network have supervised learning ability. Different from the adjustment method of matching parameter of ARTMAP network, the model in this paper gradually reduces the matching parameter, so that each type of visual place cell in the sampling region of genetic algorithm can generate unique united place cell. Meanwhile, the learned matching parameter is used for classification in the other types to generate united place cells, which makes the place cell distribution near the boundaries extend to the whole exploring space.

The spatial representation based on grid cells and place cells only implements the positioning. The cognitive map required by intelligent navigation should contain the relative relationship between independent locations and provide accurate path information for the autonomous movement of intelligent agents. Therefore, the cognitive map construction and the intelligent navigation based on the cognitive map will be the main content of our next research.

## Figures and Tables

**Table 1:** Simulation parameters of visual grid cells.

Parameter	Variable	Value
Exploring space	*L*1 × *L*2	80 m × 60 m
Exploring speed	*v*	2 m/s
Maximum firing rate of visual place cells	*A*	10 Hz
Minimum standard deviation of firing field of visual place cells	*l*_min_	1 m
Maximum standard deviation of firing field of visual place cells	*l*_max_	15 m
Maximum distance to generate visual place cell	*D*	20 m
Firing field distribution constant of visual place cells	*L*	30 m
Weight update rate	*η*	5 × 10^−6^
Reduction coefficient of firing rate of visual place cells	*h*	5
Weight update constant	*S*	7
Lower weight boundary	*Th*_down_	0
Upper weight boundary	*Th*_up_	5

**Table 2:** The simulation parameters of genetic algorithm.

Parameter	Variable	Value
Weight threshold in sampling region	Ψ	1
Population size	*N*	100
Parameter binary digit	*M*	10
Crossover probability	*pc*	0.6
Mutation probability	*pm*	0.1

**Table 3:** The firing parameters of generated united grid cells.

Boundary to generate visual grid cell	Weight update time interval	Spacing	Phase	Maximum firing rate	Direction
*y*=0	*τ*=4	16.2268	[12.2776, 29.3255]	0.9071	5.7427
		9.6970	[20.1760, 26.4516]	0.9853	6.2663
	*τ*=5	20.9189	[71.2414, 54.3109]	0.8759	5.7427
		13.7634	[27.5269, 51.0264]	0.9932	6.2663
	*τ*=6	22.9130	[37.8495, 0.5865]	0.8759	4.7477
		13.6070	[72.5709, 11.2610]	0.9932	5.2713
	*τ*=7	30.0293	[29.5601, 1.4663]	0.7341	4.7477
		17.5171	[73.5093, 48.7977]	0.9932	5.2713

*y*=60	*τ*=4	14.0371	[4.5357, 46.9795]	0.8289	4.7109
		8.3675	[61.3099, 37.0088]	0.9932	5.2345
	*τ*=5	18.4146	[38.7097, 42.7566]	0.7977	4.7109
		10.8700	[19.9413, 8.4457]	0.9853	5.2345
	*τ*=6	22.4047	[46.2170, 38.5924]	0.8133	4.6494
		13.2942	[35.3470, 44.3402]	0.9932	5.1732
	*τ*=7	28.4262	[55.5230, 58.2991]	0.7195	3.6852
		16.9306	[42.5415, 48.7390]	0.9775	4.2088

*x*=0	*τ*=4	15.5230	[14.9365, 21.5836]	0.8094	4.1888
		9.0323	[6.8035, 52.7273]	0.9971	4.7124
	*τ*=5	16.3050	[16.4223, 52.6100]	0.8358	5.2022
		9.6188	[31.1241, 11.2023]	0.9971	5.7258
	*τ*=6	23.7732	[63.8905, 54.0762]	0.8133	4.1826
		13.6070	[2.9717, 42.1114]	0.9971	4.7062
	*τ*=7	29.4819	[1.6422, 0.3519]	0.6491	6.2770
		17.5171	[20.4888, 29.4428]	0.9384	0.5174

*x*=60	*τ*=4	15.2102	[78.7488, 29.6774]	0.8133	4.1704
		9.1105	[64.3597, 22.5220]	0.9511	4.6940
	*τ*=5	20.8407	[78.1232, 41.9941]	0.8094	5.2759
		12.5122	[52.9423, 19.5894]	0.9932	5.7995
	*τ*=6	21.3881	[78.7488, 20]	0.7820	3.1877
		13.4506	[9.0714, 39.4135]	0.9951	3.7113
	*τ*=7	23.9687	[57.5562, 29.9120]	0.7830	6.2832
		14.7019	[76.5591, 16.8328]	0.9932	0.5236

## Data Availability

The data used to support the findings of this study are all available from the corresponding author upon request.
